# Assessing the sustained impact of a school-based obesity prevention program for adolescent boys: the ATLAS cluster randomized controlled trial

**DOI:** 10.1186/s12966-016-0420-8

**Published:** 2016-08-20

**Authors:** David R. Lubans, Jordan J. Smith, Ronald C. Plotnikoff, Kerry A. Dally, Anthony D. Okely, Jo Salmon, Philip J. Morgan

**Affiliations:** 1 Priority Research Centre in Physical Activity and Nutrition, School of Education, University of Newcastle, Callaghan, NSW Australia; 2 Interdisciplinary Educational Research Institute, University of Wollongong, Wollongong, NSW Australia; 3 Centre for Physical Activity and Nutrition Research, Deakin University, Burwood, VIC Australia

**Keywords:** Intervention, Fitness, Resistance training, Behavior, Disadvantaged

## Abstract

**Background:**

Obesity prevention interventions targeting ‘at-risk’ adolescents are urgently needed. The aim of this study is to evaluate the sustained impact of the ‘Active Teen Leaders Avoiding Screen-time’ (ATLAS) obesity prevention program.

**Methods:**

Cluster RCT in 14 secondary schools in low-income communities of New South Wales, Australia. Participants were 361 adolescent boys (aged 12–14 years) ‘at risk’ of obesity. The intervention was based on Self-Determination Theory and Social Cognitive Theory and involved: professional development, fitness equipment for schools, teacher-delivered physical activity sessions, lunch-time activity sessions, researcher-led seminars, a smartphone application, and parental strategies. Assessments for the primary (body mass index [BMI], waist circumference) and secondary outcomes were conducted at baseline, 8- (post-intervention) and 18-months (follow-up). Analyses followed the intention-to-treat principle using linear mixed models.

**Results:**

After 18-months, there were no intervention effects for BMI or waist circumference. Sustained effects were found for screen-time, resistance training skill competency, and motivational regulations for school sport.

**Conclusions:**

There were no clinically meaningful intervention effects for the adiposity outcomes. However, the intervention resulted in sustained effects for secondary outcomes. Interventions that more intensively target the home environment, as well as other socio-ecological determinants of obesity may be needed to prevent unhealthy weight gain in adolescents from low-income communities.

**Trial registration:**

Australian Clinical Trial Registry ACTRN12612000978864.

**Electronic supplementary material:**

The online version of this article (doi:10.1186/s12966-016-0420-8) contains supplementary material, which is available to authorized users.

## Background

Obesity is a global public health challenge and in developed countries 24 % of boys and 23 % of girls were overweight or obese in 2013 [1]. Although there is some evidence suggesting rates of pediatric obesity have levelled off in developed nations [2], this trend has not been observed in low-income communities [3]. Youth of low socio-economic position (SEP) have poorer nutritional knowledge [4] and receive less family support for physical activity and healthy eating compared with young people from middle and high socio-economic strata [4]. In addition, low-income youth are less active and spend more time engaged in sedentary screen-based recreation, compared with their higher-income peers [5]. Although the etiology of obesity is complex, these factors may partially explain why rates of obesity among low-income youth are continuing to rise.

Schools represent an ideal setting to address social inequalities because they provide access to the population and generally have the necessary facilities, curriculum, environment and personnel to promote physical activity and healthy eating [6]. School-based obesity prevention interventions targeting adolescents are particularly important as physical activity declines [7], dietary behaviors deteriorate [8] and recreational screen-time increases [9] during the teenage years. Preventing unhealthy weight gain in adolescent populations is challenging and evidence from the most recent Cochrane review of school-based obesity prevention interventions suggests that intervention effects among adolescents have been minimal (i.e., mean standardized difference in BMI/BMI *z*-score = −.09 units; 95 % Confidence Intervals [CI] = −.20 to .03). In addition, little is known regarding the sustainability of intervention effects [10, 11], as few obesity prevention programs have assessed the maintenance of improvements in adiposity and health behaviors beyond immediate posttest assessments [10].

There is clearly a need for innovative interventions that target adolescents who are ‘at-risk’ of obesity and assess the maintenance of effects over time. Previous school-based interventions have reported differential effects for boys and girls [12, 13], suggesting that males and females might benefit from more targeted intervention approaches. For example, boys may be more receptive than girls to participation in more traditionally masculine activities such as resistance training. Although muscle-strengthening activities are recommended for both boys and girls [14, 15], it is important that these physical activity preferences are recognized, particularly for interventions attempting to engage otherwise inactive youth. There are also clear and consistent gender differences in key weight-related behaviors. For example, despite being more active, boys are more likely than girls to consume unhealthy quantities of sugar-sweetened beverages (SSB) and engage in high levels of recreational screen-time [16]. These unique socio-cultural differences should inform the design and delivery of health behavior interventions for youth.

Active Teen Leaders Avoiding Screen-time (ATLAS) was an obesity prevention program targeting disadvantaged adolescent boys, considered ‘at-risk’ of obesity based on their physical activity and recreational screen-time behaviors. ATLAS was designed to be culturally appropriate and incorporated mHealth (i.e., mobile phone) technology to supplement the school- and home-based components. We previously reported null findings for changes in adiposity, but significant group-by-time interaction effects for recreational screen-time (−30 mins/d, *p* = .03), SSB intake (−0.6 glass/d, *p* = .01), muscular fitness (0.9 repetitions, *p* = .04) and resistance training skill competency (5.7 units, *p* < .001) [17] and a small positive effect for psychological well-being [18]. The aim of this paper is to report the sustained impact of the ATLAS program on primary and secondary outcomes which were assessed 10-months after program completion (i.e., 18-months post baseline).

## Methods

### Study design, setting and participants

Ethics approval for this study was obtained from the University of Newcastle, Australia and the New South Wales (NSW) Department of Education and Communities. School principals, teachers, parents and study participants all provided informed written consent. The design, conduct and reporting of this trial adheres to the CONSORT statement (see Additional file 1). The rationale and study protocol has been described in detail previously [19]. Briefly, ATLAS was evaluated using a cluster RCT conducted in state-funded secondary schools within low-income areas of NSW, Australia. The Socio Economic Indexes For Areas (SEIFA) Index of Relative Socioeconomic Disadvantage (IRSD) (scale, 1 = *lowest* to 10 = *highest*) was used to identify eligible schools. Schools located in the Newcastle, Hunter and Central Coast regions of NSW classified within an IRSD decile ≤ 5 (lowest 50 %) were considered eligible. All male students in their first year at the study schools completed a short screening questionnaire to assess their eligibility for inclusion. Students failing to meet either international physical activity (<60 mins MVPA each day) or screen-time (≥2 h per day) guidelines [15] were considered eligible and invited to participate. Randomization occurred at the school-level following baseline data collection. After baseline assessments, schools were match-paired, based on their size, socio-economic status and geographic location, and then randomly allocated to the intervention or control group using a computer-based random number producing algorithm, by a researcher not involved in the study.

### Power calculation

Assuming 80 % power, 20 % dropout and an α level of .05, it was calculated that 350 boys would be required to detect a difference between groups of 0.4 kg.m^−2^ for BMI and 1.5 cm for waist circumference [17]. The study was not adequately powered for subgroup analyses. Therefore, pre-specified subgroup analyses should be considered exploratory.

### Intervention

ATLAS was a 20-week school-based intervention [19] and included the following key components: teacher professional learning (2 × 5 h workshops); provision of fitness equipment to schools (1 × pack/school valued at ~ $1500); researcher-led seminars for students (3 × 20 min); face-to-face physical activity sessions delivered by teachers during the school sport period (20 × ~90 min, in addition to regular PE lessons); lunch-time physical activity leadership sessions run by students (6 × 20 min); pedometers for physical activity self-monitoring (17 weeks); parental strategies for reducing recreational screen-time (4 × newsletters); and a purpose-built web-based smartphone application (15 weeks). The intervention was based on Self-determination theory [20] and Social cognitive theory [21] and aimed to support students’ psychological needs for autonomy, competence and relatedness to improve their autonomous motivation for school sport and leisure-time physical activity. As national and international physical activity guidelines recommend young people engage in activity to strengthen muscles (e.g., resistance training) at least twice a week [14, 15], the intervention aimed to improve boys’ self-efficacy for resistance-based exercise, by explicitly targeting resistance training movement skill competency. Teachers were provided with professional development and equipment to deliver resistance-based exercise. These intervention components were important as muscular fitness levels of school-age youth are decreasing [22, 23] and schools and teachers often lack the necessary facilities and expertise to deliver non-traditional activities, such as resistance training [24].

The ATLAS intervention targeted adolescent boys considered to be ‘at risk’ of obesity. Although weight status was not an inclusion criteria (such an approach may lead to stigmatization and bullying), we designed the program to be appropriate for overweight youth. Resistance training is an ideal activity for overweight adolescents because they find it easier than aerobic exercise [25–27] and it can improve muscular fitness and body composition [28, 29]. In addition, resistance training has the potential to improve adolescents’ self-esteem via the mechanisms of task mastery (self-efficacy) and physical self-concept (i.e., perceived strength and appearance) [26, 30]. This is especially true among adolescent boys because power and strength appear to be aligned with male ideals of masculinity [31–33]. The ATLAS recruitment strategies were socio-culturally [34] adapted to focus on valued outcomes for young western males (e.g., “Would you like to get fitter and stronger?”). However, the intervention was carefully designed to minimize adolescents’ expectations of hypertrophy (i.e., increase in skeletal muscle size) and emphasized technical skill and competency, as recommended in pediatric resistance training guidelines [27, 35].

Teachers were trained to deliver the enhanced sport sessions using the SAAFE (Supportive, Active, Autonomous, fair and Enjoyable) teaching principles [36], designed to enhance students’ autonomous motivation for physical activity. Each session included the following structure: (i) warm up: movement-based games and dynamic stretches; (ii) resistance training skill development: resistance band and body weight exercise circuit; (iii) fitness challenge: short duration, high intensity Crossfit™-style workout [37] performed individually with the aim of completing the workout as quickly as possible; (iv) modified games: minor strength and aerobic-based games (e.g., sock wrestling, tag-style games) and small-sided ball games that maximize participation and active learning time (e.g., touch football); and (v) cool down: static stretching and discussion of ATLAS messages. Professional learning workshops and session observations were conducted to ensure that the intervention was delivered as intended and to maximize intervention impact [34].

Following the primary study endpoint (8-months), schools and participants received no further contact from the research team (except to organize data collection). However, boys continued to have access to the smartphone app. Participants in the control schools received the intervention following the 18-month assessments.

### Outcome measures

Data were collected at the study schools by trained research assistants using a standardized assessment protocol. Measures were completed at baseline, post-intervention (8-months) and 10-months post-intervention (18-months from baseline). Questionnaires were completed using an online survey with Apple iPads in exam-like conditions and physical assessments were conducted in a sensitive manner (e.g., weight and waist circumference measured out of the view of other students).

### Adiposity

The primary outcome was BMI (weight [kg]/height [m]^2^). A portable digital scale (Model no. UC-321PC, A&D Company Ltd, Tokyo Japan) and a stadiometer (Model no. PE087, Mentone Educational Centre, Australia) were used to measure weight and height and BMI-*z* scores were determine using the ‘LMS’ method [38]. Waist circumference was measured to the nearest 0.1 cm against the skin using a steel tape (KDSF10-02, KDS Corporation, Osaka, Japan) in line with the umbilicus.

### Physical activity

Actigraph accelerometers (model GT3X; Pensacola, FL, USA) were used to collect objective physical activity data. Participants’ data were included in the analyses if accelerometers were worn for ≥ 480 min per day for at least three weekdays (determined a priori). Mean counts per minute (CPM) and percentage of time in moderate-to-vigorous physical activity (MVPA) were calculated using Evenson cut-points [39].

### Sedentary behavior

A modified version of the Adolescent Sedentary Activity Questionnaire (ASAQ) [40] was used to determine time spent in screen-based recreation. To address the issue of media multi-tasking (i.e., using multiple devices concurrently), participants were asked to report their total recreational screen-time (regardless of device) for each day of the week.

### Sugar-sweetened beverage consumption

Two items from the NSW Schools Physical Activity and Nutrition Survey (SPANS) [41] were used to assess consumption of SSB’s. Students were asked to report how many glasses (one glass = 250 mL) of fruit-based drinks and soft drinks/cordial they consumed on a ‘usual’ day (range = *none* to *7 or more per day*).

### Muscular fitness

The 90° push-up test was used as a measure of upper body muscular endurance, using the protocol outlined in the Cooper Institute FITNESSGRAM® [42]. This test has acceptable test-retest reliability in adolescents (ICC [95 % CI] = .90 [.80 to .95]) [43]. A handgrip dynamometer was used to determine hand and forearm strength (SMEDLEY’S dynamometer TTM, Tokyo, Japan). The hand grip test is a valid measure of upper body maximal strength among youth and has acceptable test-retest reliability among adolescents [44].

### Resistance training skill competency

Resistance training skill competency was assessed using the Resistance Training Skills Battery (RTSB) [45]. The test requires participants to perform six movements (lunge, push-up, overhead press, front support with chest touches, squat, and suspended row) which are video recorded and scored according to predetermined criteria. An overall skill score is created by adding the six scores (possible range 0 to 56).

### Motivation for school sport

Motivational regulations for school sport were assessed using an adapted validated scale used by Goudas et al. [46]. The original items were designed for use in physical education, and were modified to assess motivation for co-curricular ‘school sport’. Five subscales were included: two autonomous (i.e., intrinsic and identified) and two controlled (i.e., introjected and external) motivational regulation subscales and one subscale measuring a lack of motivation (amotivation). According to SDT [47], autonomous motivation reflects self-determined reasons for engaging in a specific behaviour, such as experiences of enjoyment (intrinsic) or personal endorsement of the benefits to self (identified). Conversely, controlled motivation reflects the presence of externally imposed reasons for engaging in a behaviour, such as to avoid feelings of guilt or shame (introjected) or to receive rewards/avoid punishment (external). Students responded to 20 items on a 7-point scale (*1 = not at all true, 7 = very true*). Using data from the present sample, the internal consistency of items within each subscale ranged from α = .75 (introjected) to α = .85 (intrinsic).

### Analysis

Analyses followed the intention-to-treat principle and were conducted using linear mixed models in SPSS version 20.0 (IBM SPSS Statistics, IBM Corporation, Armonk, NY; 2010) with alpha levels set at *p* < .05 [48]. Mixed models assessed the impact of group (intervention or control), time (treated as categorical with levels baseline, 8- and 18-months) and the group-by-time interaction for all primary and secondary outcomes. The models were adjusted for clustering at the school level using a random intercept and participants’ SEP (defined as residential IRSD decile). Pre-specified subgroup analyses for adiposity outcomes were conducted for those classified as overweight/obese (combined as a single group) at baseline. For descriptive purposes the proportional difference between study arms among those improving their weight status (i.e., moving from “obese” to “overweight” or from “overweight” to “healthy weight”) or regressing to a poorer weight status (i.e., moving from “healthy weight” to “overweight” or from “overweight” to “obese”) is also reported.

## Results

**Fig. 1:**
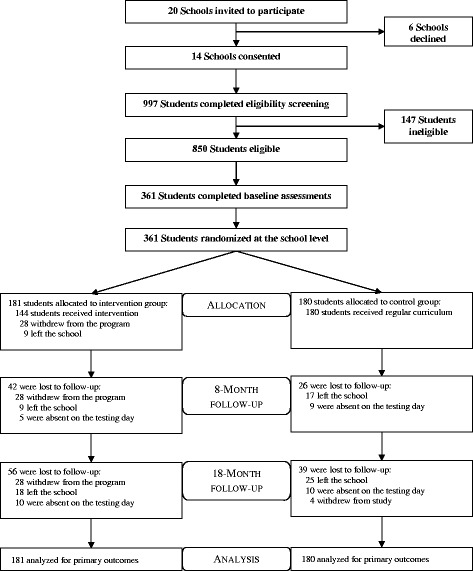
Flow of participants through the study

Fourteen schools were recruited, and 361 boys (mean age: 12.7 ± 0.5 years) were assessed at baseline (Fig. 1), satisfying the required sample size calculated a priori. Baseline characteristics of the study sample can be seen in Table 1. Briefly, the majority of boys were born in Australia, spoke English at home, and were of low- to middle SEP. In addition, approximately a third of boys were classified as overweight or obese at baseline. Posttest (8-month) and follow-up (18-months) assessments were completed for 293 (81.2 %) and 266 (73.7 %) participants, respectively. There were no meaningful differences at baseline between completers and drop-outs for primary or secondary outcomes at 18-months. Table 2 presents changes in the primary and secondary outcomes for intervention and control groups.

**Table 1:** Baseline characteristics of the study sample

Characteristics	Control (*n* = 180)	Intervention (*n* = 181)	Total (*N* = 361)
Age, mean (SD), y	12.7 (0.5)	12.7 (0.5)	12.7 (0.5)
Born in Australia, n (%)	168 (93.3)	174 (96.1)	341 (94.7)
English language spoken at home, n (%)^a^	169 (94.4)	175 (96.7)	344 (95.6)
Cultural background, n (%)^b^			
Australian	132 (73.7)	145 (80.6)	277 (77.2)
European	31 (17.3)	22 (12.2)	53 (14.8)
African	6 (3.4)	1 (0.6)	7 (1.9)
Asian	3 (1.7)	4 (2.2)	7 (1.9)
Middle eastern	2 (1.1)	0 (0)	2 (0.6)
Other	5 (2.8)	8 (4.4)	13 (3.6)
SEP, n (%)^c^			
1–2	55 (30.9)	49 (27.1)	104 (29.0)
3–4	81 (45.5)	120 (66.3)	201 (56.0)
5–6	27 (15.2)	4 (2.2)	31 (8.6)
7–8	8 (4.5)	8 (4.4)	16 (4.5)
9–10	7 (3.9)	0 (0)	7 (1.9)
Weight, kg	53.1 (13.4)	54.0 (15.0)	53.5 (14.2)
Height, cm	160.2 (8.4)	160.9 (9.0)	160.5 (8.7)
BMI, kg.m^−2^	20.5 (4.1)	20.5 (4.1)	20.5 (4.1)
Weight status, n (%)			
Thinness	5 (2.8)	2 (1.1)	7 (1.9)
Healthy weight	115 (63.9)	110 (60.8)	225 (62.3)
Overweight	38 (21.1)	39 (21.5)	77 (21.3)
Obese	22 (12.2)	30 (16.6)	52 (14.4)
Waist circumference, cm	76.5 (12.3)	76.2 (12.2)	76.3 (12.2)

Weight status was determined using the World Health Organization criteria [38]: Thinness < -2SD, Overweight: > +1SD, Obesity: > +2SD for age and sex-adjusted BMI z-scores

*Abbreviations*: *BMI* body mass index; *SEP* socio-economic position

^a^One participant did not report language spoken at home

^b^Two participants did not report cultural background

^c^Socioeconomic position determined by population decile using Socio-Economic Indexes For Areas Index of Relative Socioeconomic Disadvantage based on residential postcode (1 = lowest, 10 = highest). Two participants did not report residential postcode

**Table 2:** Changes in primary and secondary outcomes for the intervention and control groups

Outcome^a^	Baseline, Mean (SE)	n	8-month, Mean (SE)	n	Time^b^ *p*	18-month, Mean (SE)	n	Time^c^ *p*	Adjusted difference in change, Mean (95 % CI)^d^	Group-by-time^d^ *p*
Primary outcomes										
BMI, kg.m^−2^										
Intervention	20.8 (.6)	181	21.4 (.7)	139	<.001	22.3 (.7)	121	<.001	.07 (−.34, .38)	.656
Control	20.7 (.6)	180	21.3 (.6)	154	<.001	22.3 (.6)	143	<.001		
Waist circumference, cm										
Intervention	77.1 (1.9)	181	77.1 (1.9)	133	.898	78.8 (1.9)	118	<.001	.3 (−.7, 1.4)	.549
Control	77.0 (1.7)	180	76.5 (1.7)	147	.238	78.4 (1.7)	143	<.001		
Secondary outcomes										
BMI *z-*score										
Intervention	.64 (.20)	181	.66 (.20)	139	.584	.69 (.20)	121	.163	.04 (−.07, .14)	.485
Control	.51 (.18)	180	.50 (.18)	154	.761	.53 (.18)	143	.635		
Counts/min^e^										
Intervention	538 (27)	133	542 (29)	68	.830	511 (31)	46	.160	10 (−42, 61)	.715
Control	492 (24)	132	498 (25)	89	.698	455 (26)	71	.027		
Percent MVPA^e^										
Intervention	8.7 (.5)	133	4.4 (.5)	68	<.001	8.5 (.5)	46	.677	.1 (−.8, 1.0)	.805
Control	7.9 (.4)	132	4.4 (.4)	89	<.001	7.7 (.4)	71	.377		
Screen-time, min/d										
Intervention	108.0 (14.6)	180	110.7 (15.0)	137	.722	128.5 (15.2)	120	.010	−32.2 (−53.6,−10.8)	.003
Control	130.6 (13.1)	177	163.1 (13.3)	152	<.001	183.3 (13.4)	145	<.001		
SSB intake, glasses/d										
Intervention	3.9 (.4)	179	3.1 (.3)	135	<.001	4.1 (.3)	120	.414	.2 (−.4, .7)	.561
Control	3.9 (.4)	174	3.8 (.3)	152	.492	3.9 (.3)	145	.946		
Grip strength, kg										
Intervention	22.5 (.97)	181	28.5 (.98)	133	<.001	32.7 (1.0)	120	<.001	.3 (−.7, 1.2)	.580
Control	20.4 (.87)	180	25.9 (.88)	148	<.001	30.2 (.9)	143	<.001		
Push-ups (repetitions)										
Intervention	9.1 (1.0)	177	9.8 (1.0)	135	.058	11.5 (1.0)	113	<.001	.5 (−.6, 1.6)	.376
Control	6.6 (.9)	179	6.5 (.9)	148	.866	8.6 (.9)	135	<.001		
RT skill competency^f^										
Intervention	32.3 (.84)	166	40.6 (.87)	129	<.001	37.9 (.89)	107	<.001	5.9 (4.5, 7.3)	<.001
Control	30.6 (.76)	169	33.4 (.77)	145	<.001	30.4 (.78)	128	.654		
Intrinsic regulation^g^										
Intervention	6.01 (.26)	180	5.83 (.27)	135	.144	5.55 (.27)	119	<.001	.53 (.17, .88)	.003
Control	5.54 (.24)	177	5.28 (.24)	162	.024	4.55 (.24)	142	<.001		
Identified regulation^g^										
Intervention	5.94 (.24)	180	5.63 (.27)	135	<.001	5.40 (.27)	119	.016	.40 (.04, .76)	.028
Control	5.46 (.24)	177	5.12 (.24)	162	.005	4.52 (.25)	142	<.001		
Introjected regulation^g^										
Intervention	4.32 (.25)	180	4.19 (.26)	135	.267	4.54 (.27)	119	.190	.56 (.16, .97)	.006
Control	4.03 (.23)	177	3.77 (.23)	162	.047	3.67 (.24)	142	.009		
External regulation^g^										
Intervention	3.28 (.21)	180	3.51 (.24)	135	.137	4.22 (.24)	119	<.001	.49 (.05, .92)	.028
Control	3.29 (.21)	177	3.28 (.21)	162	.927	3.75 (.21)	142	.003		
Amotivation^g^										
Intervention	1.65 (.18)	180	2.04 (.19)	135	.006	2.83 (.19)	119	<.001	.13 (−.26, .52)	.511
Control	1.93 (.16)	177	2.04 (.17)	162	.384	2.97 (.17)	142	<.001		

*Abbreviations*: *BMI* body mass index; *MVPA* moderate-to-vigorous physical activity; *RT* resistance training; *SSB* sugar-sweetened beverages

^a^All models were adjusted for school clustering and participant’s household socio-economic status

^b^Within-group effect from baseline to 8-months

^c^Within-group effect from baseline to 18-months

^d^Group-by-time effect from baseline to 18-months

^e^265, 157 and 117 participants wore accelerometers for at least three weekdays at baseline, post-test and follow-up, respectively

^f^Possible values range from 0 to 56

^g^Motivation for school sport- possible values range from 1 to 7

### Changes in adiposity

At 18-months, there were no intervention effects for BMI, BMI z-score or waist circumference in the full study sample. Weight status remained stable for the majority of study participants (intervention = 85.1 % [*n* = 154]; control = 81.7 % [*n* = 147]). There was also little difference between groups for the proportion of participants who improved their weight status (intervention = 8.8 % [*n* = 16] and control = 7.2 % [*n* = 13]). However, almost twice as many control group (11.1 % [*n* = 20]) participants regressed to a poorer weight status over the 18-month study period, compared to those in the intervention group (6.1 % [*n* = 11]).

Pre-specified sub-group analyses were conducted for participants who were classified as overweight or obese at baseline (Table 3). No group-by-time interaction effects were found, but adjusted between-group differences were in the hypothesized direction. There was a within-group reduction in BMI *z*-score observed among intervention group participants (mean = −.13 BMI *z-*scores, 95 % CI = −.23 to −.03, *p* = .013), compared to a smaller reduction among those in the control group (mean = −.06 BMI *z-*scores, 95 % CI = −.16 to .05, *p* = .292).

**Table 3:** Adiposity outcomes sub-group analyses for participants who were overweight or obese (*n* = 129) at baseline

Outcome^a^	Baseline, Mean (SE)	8-month, Mean (SE)	Time^b^ *p*	18-month, Mean (SE)	Time^c^ *p*	Adjusted difference in change, Mean (95 % CI)^d^	Group-by-time *p*
BMI, kg.m^−2^							
Intervention	24.9 (.8)	25.2 (.8)	.167	26.1 (.8)	<.001	−.27 (−.93, .39)	.414
Control	25.4 (.7)	26.2 (.7)	.002	26.9 (.7)	<.001		
BMI *z-*score							
Intervention	1.93 (.17)	1.81 (.17)	.015	1.80 (.17)	.013	−.08 (−.22, .07)	.305
Control	1.96 (.14)	1.93 (.14)	.540	1.91 (.14)	.292		
Waist circumference, cm							
Intervention	89.7 (2.9)	88.5 (2.9)	.111	89.1 (2.9)	.413	−1.2 (−3.4, 1.0)	.276
Control	91.0 (2.4)	90.4 (2.4)	.406	91.7 (2.4)	.468		

*Abbreviations*: *BMI* body mass index

^a^All models were adjusted for school clustering and participant’s household socio-economic status

^b^Within-group effect from baseline to 8-months

^c^Within-group effect from baseline to 18-months

^d^Group-by-time effect from baseline to 18-month

### Changes in behavioral outcomes

There was a group-by-time interaction effect for recreational screen-time (mean = −32.2 mins/day, 95 % CI = −53.6 to −10.8, *p* = .003). However, there were no intervention effects for physical activity or SSB consumption.

### Changes in fitness and skill outcomes

The intervention effects for muscular fitness (hand grip and push-ups) were not sustained after 18-months. However, sustained improvements were found for resistance training skill competency (mean = 5.9 units, 95 % CI = 4.5 to 7.3, *p* < .001).

### Changes in motivation for school sport

There were group-by-time interaction effects for intrinsic, identified, introjected and external regulations in favor of the intervention group (range for adjusted difference between groups = .40 to .56, *p* < .05 for all). There was no intervention effect for amotivation for school sport, which increased for both groups over the study period.

## Discussion

The aim of this paper was to report the sustained impact of the ATLAS intervention on adiposity, fitness and health behaviors in a sample of adolescent boys attending schools in low-income communities. Contrary to our primary hypothesis, the ATLAS intervention had no immediate or sustained impact on adiposity. There was some support for a positive effect among participants who were overweight or obese at baseline, as demonstrated by a reduction in BMI *z*-score within the intervention group. However, the effect is unlikely to be clinically meaningful and may reflect fluctuations in adiposity not attributable to the intervention. Our null findings are consistent with previous obesity prevention interventions targeting adolescents, which have been less successful than interventions targeting children [10]. Such findings may highlight the need for earlier intervention to prevent obesity among youth.

ATLAS was an obesity ‘prevention’ program and participants were considered to be ‘at-risk’, based on their physical activity and screen-time behaviors. Although the inclusion of BMI and waist circumference criteria may have increased our ability to identify and target overweight and obese adolescents, this approach was considered unacceptable by the NSW Department of Education, due to concerns that participants may experience stigmatization. Consequently, only 35 % of the study sample was overweight or obese, thus limiting our capacity to detect meaningful changes in adiposity. While our null findings for the primary outcomes are disappointing, we were successful in recruiting and retaining more than 100 overweight/obese adolescent boys, who benefited from participating in the program in other ways (e.g., improvements in screen-time, movement skills, motivation etc). Of note, community-based obesity treatment programs often report difficulty recruiting and retaining overweight youth [49, 50].

Part of the challenge in evaluating obesity prevention interventions is the selection of the most appropriate outcome measure. Although BMI is considered a good measure of adiposity change in growing children [51], it may lack sensitivity in intervention trials conducted with adolescents. Peak height velocity typically occurs during early-to-mid adolescence and it is likely that such drastic changes in height and weight masks intervention effects. A similar intervention study with adolescent boys recently reported improvements in multiple adiposity variables measured using Dual-Energy X-ray Absorptiometry (DEXA) following 8 weeks of resistance and interval training [52]. Unlike BMI, which does not distinguish between mass from different body tissues, DEXA allows researchers to determine changes specifically in fat mass. Considering weight-bearing exercise can increase adolescents’ muscle and bone mass [53], it is possible that BMI lacked validity as a measure of change in adiposity in the present trial.

The ATLAS intervention was not successful in minimizing the decline in physical activity that occurs during adolescence. Although it should be noted that compliance with accelerometer protocols was poor, making it difficult to draw any firm conclusions regarding changes in physical activity. Evidence suggests that socio-ecological interventions providing opportunities for young people to be active in different domains within and beyond the school day are needed [54, 55]. For example, the recent Physical Activity 4 Everyone (PA4E1) trial was successful in promoting physical activity and preventing unhealthy weight gain among disadvantaged adolescents [56]. However, it is worth noting that moderator analyses revealed a lack of improvement among low-active adolescents and those who were overweight or obese at baseline. Such findings suggest ‘whole-of-school’ programs may need to be supplemented with targeted programs for the most vulnerable youth.

Longitudinal research has demonstrated that motivation for physical activity declines during adolescence [57]. While ATLAS was successful in preventing a decline in autonomous motivation (intrinsic and identified), controlled motivation increased among participants in the intervention group, with no effect on amotivation. The increase in controlled motivation observed in the current study was an unintended outcome. However, controlled regulation and particularly introjected regulation can be considered a transition phase between amotivation and autonomous motivation [58]. Structured learning environments, such as the ATLAS sports sessions, where teachers communicate clear expectations, offer challenging tasks, and provide supportive and positive feedback, foster students’ self-determined forms of extrinsic motivation for physical activity and physical education [58–60]. The ATLAS boys’ higher participation in, and satisfaction with, the teacher-directed sessions as opposed to the student-directed sessions suggest that these adolescent boys were still largely motivated by the presence, or pressure, of their teachers. A gradual fading of teacher control in the sports sessions may help to reduce this reliance on external control, while teacher guidance during the initial stages of the lunchtime peer sessions may help to increase student’s feelings of competence and enhance their intrinsic motivation.

Although controlled behavioral regulations are typically considered unfavorable, within the physical activity context changes to these constructs may not be universally negative. A recent systematic review found that both autonomous and controlled behavioral regulations were associated with physical activity in young people [61], albeit that the strongest associations were for intrinsic motivation. However, Gillison and colleagues [57] demonstrated that identified rather than intrinsic regulation was the strongest predictor of exercise maintenance over 10 months in a sample of adolescent boys. This finding suggests that exercise engagement in adolescent boys may be determined more so by the perceived importance of the activity, rather than by inherent enjoyment. The same study also found that introjected regulation was a predictor of exercise in adolescent boys [57]. Although introjected and external regulations are considered less ‘self-determined’ forms of motivation, enhancing these regulations may still be beneficial assuming improvements in autonomous forms of motivation are also present and maintained, as observed in the current study.

Intervention effects for physical activity and fitness are often not maintained over the long term in school-based trials, whereas improvements in movement skill competency appear to have greater sustainability [62]. In the present trial, the group-by-time effects for muscular fitness at 8-months were not sustained. Consistent with the principle of reversibility (i.e., the loss of fitness gains following cessation of training), it is likely that boys no longer participated in sufficient muscle strengthening physical activity to sustain fitness gains. Further increases in muscular fitness over time were most likely due to normal growth and maturation.

In contrast to the fitness outcomes, improvements in resistance training skill competency were sustained at 18-months. Stodden and colleagues have described the important role of movement skill competency as a foundation for future physical activity [63]. However, skill competency has typically been operationalized as competency in fundamental movement skills (FMS) (e.g., running, jumping, catching etc). While FMS are clearly important [64], young people require a diverse set of movement skills to be physically active throughout the lifespan [65]. Developing resistance training skills may provide individuals with the confidence and competence to engage in muscle strengthening activity, as recommended in national physical activity guidelines. We previously demonstrated that improvements in resistance training skills mediated the effects of ATLAS on muscular fitness and body fat at 8-months [66], suggesting that explicitly targeting movement skills might help to improve these outcomes.

Reducing screen-time was a key behavioral target in the ATLAS intervention, which was designed to enhance adolescents’ autonomous motivation to limit their screen-time (i.e., personally valuing the benefits of limiting screen-time). In regards to this outcome ATLAS was successful, with a sustained between-group difference of 32 mins/day at 18-months. Interventions to reduce screen-time have had mixed results and the majority of previous interventions have targeted children [67]. A recent meta-analysis of nine screen-time interventions conducted with children and adolescents reported an average between-group difference of just −.90 h/week (95 % CI, −3.47 to 1.66 h/week, *p* = 0.494) [67], or approximately eight minutes per day. Notably, this is a substantially smaller effect than that observed in the current study.

The study strengths include the cluster RCT design, longer-term follow-up to assess maintenance of intervention effects, as well as high rates of retention and intervention fidelity (see main outcomes paper for details [17]). The potential scalability of this program is another notable strength, which is demonstrated by interest from key stakeholders in the education system. Following the completion of the ATLAS RCT, the research team was asked by the State of New South Wales (NSW) Department of Education to refine the intervention for dissemination in NSW secondary schools. The intervention has subsequently been modified to enhance sustainability and scalability by: i) removing the pedometer and parental newsletters, ii) reducing professional development from two days to one day, and iii) reducing program duration from 20-weeks to 10 weeks. ATLAS version 2.0 is currently being evaluated in a nationally-funded, cluster RCT and dissemination study [68].

Despite these strengths, it is important to note some limitations. First, we do not have objective usage data to determine students’ on-going engagement with the smartphone app. Second, compliance with accelerometer protocols was poor and only 32 % of participants were included in the analysis for this outcome. This finding is not surprising, as study participants were low-active adolescent boys attending schools in low-income communities. A population-based cohort study of young people’s accelerometer-determined activity (*N* = 13,681) found that non-compliers to accelerometer protocols were more likely to be: i) male, ii) overweight/obese, iii) inactive, and iv) low-SEP [69]. Although we used procedures and incentives previously shown to enhance monitoring compliance among adolescents [70], these strategies appeared to be ineffective with our study population. Finally, our drop-out rate of 26 % (at 18-month assessments) was higher than anticipated. Drop-out rates vary considerably in school-based obesity prevention studies [71], with higher drop-out rates typically observed among adolescents attending schools in disadvantaged communities (e.g., [72, 73]). However, it is difficult to compare our findings with similar studies because few obesity prevention studies include longer-term follow-up assessment after initial intervention delivery [11].

## Conclusions

We can conclude the intervention was not successful in its primary aim of obesity prevention. The maintenance of intervention effects for screen-time, resistance training skill competency and motivation for school sport suggest that the intervention was successful in producing positive and sustained effects for these outcomes. Adolescents most at risk of adverse health outcomes (i.e., overweight and low SEP youth) are often the least likely to benefit from broad ‘whole-of-school’ interventions. Our findings demonstrate the potential for school-based programs to provide ‘at-risk’ adolescents with behavioral (e.g., goal setting and self-monitoring) and movement skills (i.e., resistance training skills) using a targeted program. However, interventions that more intensively target the home environment as well as other socio-ecological determinants of obesity are most likely needed for the successful prevention of unhealthy weight gain among this population.

## Additional file

Additional file 1: CONSORT checklist. (DOCX 23 kb)

## Abbreviations

ASAQ: Adolescent sedentary activity questionnaire
ATLAS: Active teen leaders avoiding screen-time
BMI: Body mass index
CI: Confidence intervals
CONSORT: Consolidated standards for reporting trials
CPM: Counts per minute
DEXA: Dual-energy x-ray absorptiometry
FMS: Fundamental movement skills
ICC: Intra-class correlation coefficient
IRSD: Index of relative socio-economic disadvantage
MVPA: Moderate to vigorous physical activity
NSW: New South Wales
RCT: Randomized controlled trial
RTSB: Resistance training skills battery
SDT: Self-determination theory
SEIFA: Socio-economic indexes for areas
SEP: Socio-economic position
SPANS: Schools physical activity and nutrition survey
SSB: Sugar-sweetened beverages
